# From Trauma to Resilience: A Systematic Review of Psychological Intervention Strategies for War-Affected Children and Adolescents

**DOI:** 10.1055/s-0046-1819570

**Published:** 2026-04-16

**Authors:** Raghad H. Alkhalifah, Bana AlBani, Nasser E. Alotaibi, Waleed D. Khubzan, Reema M. Alharbi, Abdulhadi J. Alotaibi, Reema Alsweed, Abdullah Alqifari

**Affiliations:** 1 College of Medicine, Qassim University, Qassim, Saudi Arabia; 2 College of Medicine, AlMaarefa University, Riyadh, Saudi Arabia; 3 College of Medicine, Prince Sattam bin Abdulaziz University, AlKharj, Saudi Arabia; 4 College of Medicine, Taif University, Taif, Saudi Arabia; 5 Department of Medicine, Vision Colleges, Riyadh, Saudi Arabia; 6 Department of Psychiatry, College of Medicine, Qassim University, Buraydah, Saudi Arabia

**Keywords:** war, PTSD, trauma

## Abstract

Globally, 1 in 10 children are impacted by war, with over 24,000 violations documented in 2020 alone. These children face persistent mental health challenges, including anxiety, depression, and posttraumatic stress disorder (PTSD). This systematic review evaluates current psychological interventions for war-affected children. Following PRISMA guidelines, 10 studies (2013–2021) from conflict zones in non-high-income countries were analyzed. Clinical interventions such as the Teaching Recovery Techniques (TRTs) are crucial; TRT is a low-cost intervention implementable by trained nonprofessionals under supervision. Findings revealed that TRT showed promise, especially when combined with parenting sessions, underscoring the importance of caregiver involvement. School-based programs were effective in addressing PTSD and anxiety symptoms, highlighting the key role of schools and trained teachers. Reading interventions improved affect recognition, and life-skills approaches enhanced social coping, but the most impactful strategies integrated caregivers. Successful interventions combined parental and community support, emphasizing cultural adaptation and individualized approaches for sustained mental health improvements.

## Introduction

Children living in war-affected regions suffer severe challenges that impact their health, safety, and overall well-being. In 2020 alone, over 24,000 grave violations against children were documented, and globally, 1 in 10 children are affected by armed conflicts. Exposure severity and recency significantly increase the risk of posttraumatic stress disorder (PTSD) and other mental health problems. 1 2 3 4 The number and type of war traumas experienced by children vary based on factors such as age, gender, and parental socioeconomic status. 5 Unaccompanied refugee minors are especially vulnerable, with separation from family, death of loved ones, and exposure to armed conflicts being common stressors. 6

Bereavement can lead to heightened levels of depression, while parental separation can impair prosocial behavior. 5 This highlights the need for supportive, community-based interventions. 7 For instance, studies in Afghanistan and Sri Lanka show that family violence often co-occurs with war trauma. 7

There is growing emphasis on strengths-based approaches that support children's resilience and build on existing community resources. 8 This study examines intervention strategies for war-affected children, focusing on psychological, physical, and nutritional outcomes. It evaluates current psychological interventions and identifies effective practices for program implementation. The ultimate objective is to support practitioners working in conflict-affected settings by offering evidence-based recommendations that enhance health outcomes for children and help foster resilience in the face of unimaginable adversity. By considering the interplay between psychological well-being and physical health, this review adopts a holistic perspective, recognizing the interconnected nature of these domains in the context of trauma recovery.

## Methods

### Study Design and Setting

This systematic review followed the Preferred Reporting Items for Systematic Reviews and Meta-Analyses (PRISMA). The study protocol was registered in PROSPERO (CRD42024574094).

### Outcomes

This systematic review aims to evaluate the effectiveness and response of different psychological treatment methods.

### Research Strategy

This research included 10 studies that were conducted through searches in PubMed, Cochrane, and Google Scholar (2013–2021). The selected keywords and terms include “war-affected children” OR “children in conflict zones” OR “child war survivors” OR “children affected by war” with “ intervention strategies” OR “psychosocial intervention” OR “therapeutic intervention” OR “counseling” OR “education programs” OR “community support” OR “therapeutic activities” with “resilience” OR “mental health” OR “psychological wellbeing” OR “social adjustment” OR “emotional resilience” OR “mental resilience.”

### Inclusion and Exclusion Criteria

Studies included were randomized controlled trials (RCTs), quasi-experimental studies, pre-post studies, retrospective studies, and prospective studies published in English, evaluating psychosocial interventions for children in conflict-affected low- and middle-income countries. Studies not focused on armed conflict, from high-income countries, or without an evaluation component were excluded.

### Data Extraction and Screening

Titles and abstracts were screened independently by two reviewers using Rayyan software. Full texts meeting criteria were reviewed. Disagreements were resolved with a senior author.

### Risk of Bias Assessment

Data were extracted using a standardized form, and study characteristics were recorded. The methodological quality of included studies was assessed by one individual using the Cochrane risk of bias (RoB) tools. 9 10 11 Four studies 12 13 14 15 were evaluated using RoB 2.0, 9 two studies 16 17 with the RoB 2.0 cluster-RCT tool, 10 and the remaining four studies 18 19 20 21 were assessed using the risk of bias in nonrandomized studies—of interventions (ROBINS – I) tool. 11

## Results

### Study Selection and Participant Characteristics

**Fig. 1:**
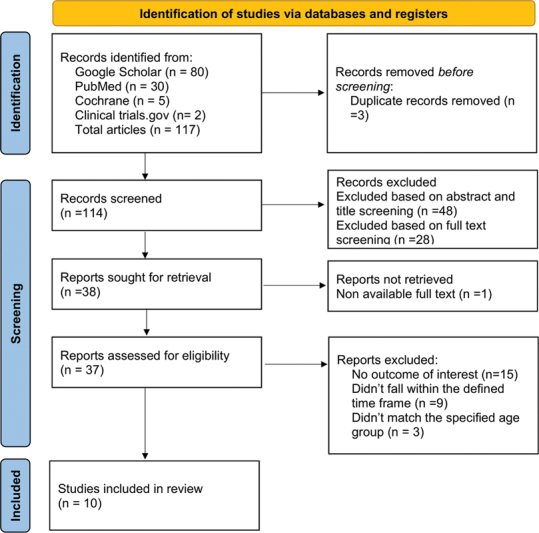
PRISMA diagram showing number of studies identified, screened, and included in the final review.

A total of 117 articles were retrieved, with 10 studies meeting the inclusion criteria ( Fig. 1 ). The included studies are summarized in Table 1 . Eight were RCTs, one was a nonrandomized trial, and one cross-sectional study. The studies took place in multiple regions, including Palestine, Jordan, Turkey, Lebanon, Syria, Burundi, and Sudan.

**Table 1:** Summary of the selected studies

Author	Year	Country	Study design	Intervention	Duration of the study	Sample size	Age range	Providers	Data collection methods	Results: Key findings related to effectiveness
El-Khani et al 12	2021	Lebanon	RCT	Combination of TRT and parenting skills training (TRT + P) focused on improving the parents' ability to support their children's emotional and psychological recovery from trauma.	Data were collected at baseline and 2 weeks and 12 weeks post-intervention.	119 (from 262 CRIES-13+ screened).	9–12 years	Local research assistant who had been given training in procedures remotely.	- Self-report: CRIES-13 (child PTSD symptoms). - Interviews: Qualitative data from children/caregivers. - Group sessions: Baseline/follow-up measurements. - Remote methods: Online/phone surveys (COVID-19 adaptation).	- PTSD Symptoms: CRIES-13 identified clinical PTSD levels; TRT + parenting skills reduced symptoms versus standard care, improving emotional regulation. - Mental Health: Anxiety/depression were assessed to evaluate intervention impact. - Parenting skills: Enhanced trauma-responsive caregiving and emotional support. -Well-being and coping: Improved child–caregiver emotional communication and stress-management strategies.
Punamäki et al 13	2014	Palestine	RCT	TRT, based on CBT, involved the safe place method, sharing and drawing frightening experiences, and dream work. Also included problem-solving, storytelling, and role-play techniques.	9 months approximately	( *N* = 482), intervention group ( *n* = 242), control group ( *n* = 240)	10–13 years (mean age = 11.29, SD = 0.68)	Two male and two female psychologists trained in CBT techniques and had experience working with children affected by war and their families.	By Self-Report Questionnaires, which are: Emotion Regulation Questionnaire, Depression Self-Rating Scale CRIES, SDQ, MHC-SF.	- TRT did not improve emotion regulation (ER) but reduced symptom intensity (↓ PTSS, depression, distress; ↑ well-being). - ER did not mediate mental health outcomes or improve functional strategies.
Diab et al 14	2014	Palestine	RCT	Teaching recovery techniques	6 months approximately	482	10–13 years Mean = 11.29	Two female and two male counsellors.	By six research assistants. Psychosocial well-being by The Mental Health Continuum–Short Form. Prosocial behavior by the SDQ. Maternal attachment by the 10-item measure of Willingness to Serve as a Secure Base for the Child. Family atmosphere was measured by the Family Ambiance Scale.	The intervention group showed a statistically significant decrease in prosocial behavior at 6 months. The intervention failed to improve psychosocial well-being or prosocial behavior as intended.
Diab et al 16	2013	Palestine	Cluster RCT	The TRT intervention involved psychoeducation, coping skill training, emotional regulation techniques, and symbolic/verbal processing of trauma through narrative, drawing, and relaxation.	The study was conducted across three assessment points: -Baseline (T1). - Post-intervention 2 months later (T2). - Follow-up 6 months post-intervention (T3). Thus, the total duration of the study was approximately 8 months	423	10–13 years	The intervention was delivered by four counselors (two male and two female), all of whom had a master's degree in psychology and were trained in counseling, including the TRT techniques. The intervention fidelity was ensured through weekly supervision by one of the study's authors.	Structured questionnaires and scales, including the Children's Revised Impact of Event Scale, Strengths and Difficulties Scale, Depression Self-Rating Scale, and Mental Health Continuum–Short Form.	- Enhancing emotional regulation and coping skills. - Addressing traumatic memories through controlled and integrated processing. - Facilitating self-expression and disclosure of traumatic experiences.
Tol et al 17	2014	Burundi	Cluster RCT	Classroom-based intervention with cognitive-behavioral techniques (e.g., psychoeducation, coping skills training) and creative expressive elements (e.g., drama, music, drawing).	October 2006–June 2007 (intervention spanned 5 weeks, with follow-up at 3 months)	329 children (153 intervention, 176 control)	8–17 years,	Locally trained paraprofessionals (nonspecialized facilitators)	Standardized and culturally adapted measures, including the Child PTSD Symptom Scale and the Children's Hope Scale, among others.	- No main effects of the intervention on primary outcomes (PTSD, depression, anxiety). - Moderators influenced outcomes, such as household size and family composition. - Some benefits were seen in younger children, those with lower trauma exposure, and those living with both parents.
Eiling et al 18	2014	Republic of South Sudan	Mixed-method, nonrandomized, pre- and post-test exploratory evaluation.	“I DEAL” life skills intervention focused on strengthening resilience through participatory activities (e.g., role-play, drawing, and games).	Intervention lasted 4–6 months, with evaluations conducted in April and November 2012.	122 children participated in the evaluation, with additional interviews conducted with 7 teachers, 11 parents, and 5 facilitators.	8–16 years	Locally trained community workers (facilitators).	- Well-being exercises to align the intervention with local perceptions of well-being. - Personal goals measured on a visual scale. - Interviews and group discussions with children, teachers, and parents.	- Reduced aggression and conduct issues. - Improved emotional coping skills and decreased worries. - 48% of children reported significant personal improvement. - Enhanced relationships and reduced fighting. - Improved school performance due to increased confidence and respect.
Gormez et al 19	2017	Turkey	Prospective experimental intervention with a pre-test/post-test design and without the use of a control group	Effectiveness of the intervention was evaluated by a pre-test/post-test comparison using the CPTS-RI, SCAS, and SDQ and CBT	8 weeks (between April 2015 and July 2015)	A total of 32 participants, mostly females, were randomly selected from a sample of 113	10–15 years	Teachers trained by the study team to deliver a weekly	Pre-test/post-test design using standardized tools: - CPTS-RI for trauma-related symptoms. - SCAS to measure anxiety. - SDQ for behavioral and emotional functioning. - Fidelity assessment through video recording and analysis of teacher-delivered sessions to ensure adherence to the intervention protocol.	The intervention reduced PTSD (CPTS-RI) and anxiety (SCAS) posttreatment, and improved short-term emotional/behavioral scores (SDQ), though effects diminished by follow-up.
Michalek et al 20	2021	Jordan	Pilot study, longitudinal design with baseline, post-intervention, and follow-up measurements.	Type: A reading-based intervention called We Love Reading, which is a nonmediated reading program aimed at improving emotion recognition and mental health through socialization.	5 months	Total participants: 94 (49 Syrian refugee children and 45 Jordanian non-refugee children).	7–12 years old, with a mean age of 8.9 years (SD = 1.3).	The intervention was delivered by trained local women (referred to as We Love Reading ambassadors) and teachers. These women were trained over a 2-day period to read stories aloud to children.	Quantitative Measures: - Emotion recognition tasks: Children classified facial expressions morphed between two emotions (e.g., happy–sad, fear–anger). - Mental health surveys: Locally validated questionnaires were used to assess optimism, depression, anxiety, distress, and insecurity. - Longitudinal design: Data collected at three time points (T1, T2, and T3).	Emotion recognition: - Initial sadness bias improved post-intervention but reverted at follow-up. - No fear-anger bias detected. Mental Health: - No significant reductions in depression/anxiety. - Refugee children showed poorer baseline mental health versus non-refugees.
Veronese et al 21	2021	Syrian refugee children in Jordanian refugee camps	Quantitative, cross-sectional discriminant analysis	Not applicable (study focused on analysis rather than intervention)	Conducted over 2 months in July–August 2014	311 children	7–14 years	Social workers and trained educators conducted assessments.	Quantitative self-report measures (e.g., SDQ) to assess participants' exposure to trauma, individual levels of resilience and mental health.	- High-resilience children exhibited less trauma symptoms (re-experiencing/hyper-arousal/avoidance) and less emotional distress. - Low-resilience children showed high trauma symptoms and increased distress. - Protective factors: Prosocial behavior (+ social relationships) strongly correlated with resilience.
Miller et al 15	2020	Lebanon	Pilot RCT	A nine-session group intervention aimed at improving caregiver stress, psychosocial well-being, and parenting practices. Included stress management techniques, relaxation activities, and positive parenting methods.	20 months, November 2017 to July 2019	78 families (151 parents)	3–12 years	Nonspecialist facilitators trained by War Child Holland, supervised by social workers, and supported by local psychologists.	Parenting: - Parenting Scale (24 items) - Parenting Knowledge Scale (15 items) Caregiver Measures - Caregiver Stress Scale (8 items) - WEMWBS (Well-being) (14 items) - K10 (Psychological Distress) (10 items) - Stress Management Scale (10 items) Child Measures - Parent-Report Kid/Kiddy-KINDL (24 items) - Child Self-Report Kid-KINDL (24 items)	Parents: - Significant improvement in psychosocial well-being, reduced psychological distress, and improved stress management. Children (as reported by parents): - Increased child psychosocial well-being. - No PTSD symptoms, anxiety, or depression were explicitly measured. Caregiver Support Intervention showed promising results for strengthening parenting in refugee communities.

Abbreviations: CPTS-RI, Child Post-Traumatic Stress Reaction Index; CRIES, Children's Revised Impact Event Scale; CBT, cognitive-behavioral therapy; COVID-19, coronavirus disease 2019; DSRS-C, Depression Self-Rating Scale for Children; ER, emotion regulation; I DEAL, War Child Holland's psychosocial support intervention; MHC-SF, Mental Health Continuum-Short Form for Youth; PTSD, post-traumatic stress disorder; PTSS, post-traumatic stress symptoms; RCT, randomized controlled trial; SCARED, screen for childhood anxiety-related disorders; SCAS, Spence Children's Anxiety Scale; SDQ, Strengths and Difficulties Questionnaire; TRT, teaching recovery technique; TRT + P, TRT + parenting; T1, baseline; T2, post-intervention 2 months later; T3, follow-up 6 months post-intervention.

The total number of participants was 2,478 (males = 1,170, 47.2%; females = 1,308, 52.8%), their ages ranged from 7 to 17 years, and the duration of the studies ranged from 1 to 20 months, with a mean of 6.2 months. The interventional studies had providers who were either professional psychologists or trained teachers to evaluate the children.

### Risk-of-Bias Assessment

**Fig. 2:**
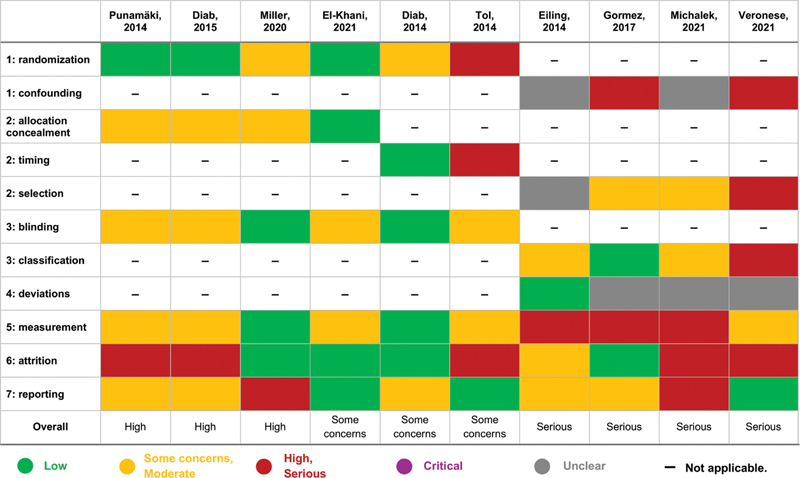
Risk of bias assessment for the 10 included studies. The matrix evaluates methodological quality across key domains, including randomization, confounding, allocation concealment, blinding, and attrition. Risk levels are color-coded: green indicates low risk of bias; yellow represents some concerns/moderate risk; red denotes high/serious risk; purple signifies critical risk; and gray marks unclear risk of bias. Dashes (–) indicate domains that were not applicable to particular study designs.

The risk of bias was assessed using RoB 2, RoB 2 for cluster RCTs, and ROBINS-I tools ( Fig. 2 ). Among RCTs, assessments ranged from low to high risk, with concerns in domains such as allocation concealment and blinding. Studies assessed with ROBINS-I showed serious overall risk, primarily due to confounding and selection bias.

### Interventions and Techniques

Multiple interventions were used to achieve better mental health for the children. Four studies used the Teaching Recovery Technique (TRT) intervention, a trauma-focused intervention involving psychoeducation, relaxation training, trauma narration, cognitive reconstruction, social support, and problem-solving, typically delivered over five to seven sessions, each lasting about 1 to 2 hours. It was developed by Patrick Smith, William Yule, and others. 22 Two studies evaluated school-based interventions, through specially designed lectures, role-plays, group discussions, and other strategies using group activities. Other approaches included were War Child Holland's psychosocial support intervention (I DEAL), a reading-based intervention, and Caregiver Support Intervention.

### Outcomes Assessment

Different scales were used across the studies to assess different psychological issues like PTSD, anxiety, depression, anger, and post-traumatic stress symptoms.

PTSD and related symptoms were assessed using the Children's Revised Impact of Event Scale (CRIES) 23 and Child Post-Traumatic Stress Reaction Index (CPTS-RI).

Depression was measured with the Depressive disorder on Self-Rating Scale for Children (DSRS-C); anxiety disorder with the Screen for Childhood Anxiety-Related Disorders (SCARED) and Spence Children's Anxiety Scale (SCAS) 24 25 ; psychological distress with the Strengths and Difficulties Questionnaire (SDQ) 26 ; and mental health with Mental Health Continuum-Short Form for Youth (MHC-SF). 27

### Teaching Recovery Technique Intervention

TRT outcomes varied across studies. Diab et al reported that TRT over 8 months improved peer relations and yielded partial mental health improvement compared with a waitlist control. 16 Punamäki et al found that TRT reduced the intensity of the emotion regulation (ER) problems, though it did not improve the overall ER capacity. 17 Crucially, El-Khani et al demonstrated that TRT augmented with parenting sessions over 3 months led to significantly greater reductions in the children's depression, anxiety, and stress when compared with the standard TRT or waitlist control groups. 12 Conversely, Diab et al found 6 months of TRT to only mildly improve general resilience. 14

### School-Based Interventions

Gormez et al found that a teacher-led, school-based program significantly reduced PTSD symptoms and anxiety in the short term. However, there were no significant long-term effects observed at follow-up. 19 Similarly, Tol et al reported that a 5-week school-based intervention did not improve stress or depressive symptoms, suggesting limited sustained impact without further support. 17 The authors believe the intervention to have preventive benefits rather than treatment.

### Life Skills and Other Interventions

The I DEAL life skills program enhanced social coping; 48% of children reported significant personal improvement (e.g., less aggression, better relationships), while 22% reported no change. 18 A reading-based intervention initially improved emotion recognition, but these gains were not maintained at a 2-month follow-up. 20

## Discussion

This review identifies several evidence-based psychological interventions for war-affected traumatized children with great emphasis on structured, trauma-focused approaches. Key among these is the TRT program, a crucial intervention, notable for its minimal costs and potential for implementation by trained nonprofessionals under the supervision of a psychologist or psychotherapist. Evidence shows that TRT combined with parenting sessions results in clearer reductions in PTSD, anxiety, and depression than TRT alone, highlighting the value of strengthening trauma-responsive caregiving. 12 The involvement of parents is therefore critical to achieve better results. School-based group programs can offer short-term improvements in PTSD and anxiety when delivered by trained teachers, underscoring the vital role of schools, although sustained effects require continued support. 19 Life-skills models such as the I DEAL program improve coping, reduce aggression, and enhance social functioning by using participatory activities aligned with local perceptions of well-being. 18

The TRT was one of the most frequently studied interventions with variable outcomes. For instance, El-Khani et al determined that the augmentation of parenting sessions with TRT was associated with a significant reduction in depression, anxiety, and stress in children in comparison to standard TRT or control conditions. 12 Other studies provided less consistent results. Diab et al found no significant gain in children's resilience over the period despite receiving TRT for 6 months. 14 In the same manner, Punamäki et al documented that although TRT was significant in decreasing the severity of emotional regulation issues, its impact was limited in facilitating general emotional resilience. 13 Such discrepancies may be due to variations in implementation settings, highlighting the importance of adapting implementation to local contexts and ensuring adequate follow-up in clinical practice..

Although with limitations, other interventions, such as school-based programs, showed promise. Elaborating on school involvement, Gormez et al found that a teacher-led, school-based intervention significantly reduced anxiety, behavioral functioning, and PTSD symptoms in the short term. 19 However, as demonstrated by Tol et al, who discovered little effect on stress and depression symptoms following 5 weeks of intervention, these advantages did not last for very long, suggesting the need for embedded, ongoing support within educational systems. 17 Children who participated in life skills programs like I DEAL reported better social coping and less aggression, highlighting yet another aspect of their efficacy. Nearly half of the participants reported personal growth, while 22% reported no change, according to Eiling et al, 18 suggesting variation in the effectiveness of the intervention.

The intricacy of treating trauma in children is further demonstrated by the testing of reading-based interventions by Michalek et al. 20 While the intervention initially improved emotion recognition and reduced sadness, these gains dissipated 2 months post-intervention, indicating a need for sustained support. This aligns with broader evidence suggesting that while many interventions provide immediate relief, long-term efficacy often requires additional components, such as active caregiver involvement or ongoing follow-ups integrated with community and school structures.

These findings have implications for a multi-dimensional and sustainable psychological practice for war-affected children. Evidence has indicated that combining techniques, such as TRT with parental support or integrating school-based programs with community involvement, optimizes outcomes. Thus, contextualized interventions that consider different cultural contexts and individual child needs would be critical for achieving long-term mental well-being benefits. This study contributes to the growing body of evidence advocating holistic, evidence-based strategies to support children in conflict zones effectively and responds to the huge need for clinically focused guidance.

## Limitations

Although this study provides a powerful insight into interventions used for children affected by armed conflicts, it also has several limitations that must be mentioned: (1) limited sample size: only 10 studies were included, with a total sample size of 2,478 participants. This is considered a small sample size, especially when assessing the optimal interventions used across multiple regions. (2) Study designs variability: the variability of study designs increased the heterogeneity, making it difficult to draw a uniform conclusion. (3) Data collection challenges: collecting data in armed conflict areas is difficult due to several reasons, such as difficulty in communicating with participants, lack of a convenient environment, and weak capabilities of the institutions. (4) High risk of bias: many of the included studies have a high and serious risk of bias, which might have affected the reliability of the outcomes. (5) Follow-up periods: most of the included studies showed short follow-up after the interventions and lacked the long-term effect of the interventions. (6) Security concerns: the security situation in the areas where studies were conducted might have affected both researchers and participants. Those security concerns could have led to restricting the population research and affecting the intervention outcomes indirectly.

Future studies must acknowledge and avoid these limitations. More rigorous designs should be made to minimize the risk of bias. Meta-analysis could be applied in future studies once sufficient similar studies with close techniques are available to provide a clearer and more robust understanding of the effectiveness of the interventions statistically.

## Conclusion

This review identifies TRT, particularly when combined with caregiver-focused components, as the intervention showing the most consistent improvements. For clinical practice, the best practices emerging from the evidence are: (1) structured, trauma-focused interventions like TRT, delivered by trained nonspecialists, (2) school-based programs that utilize trained teachers, and (3) life skills training that enhances social coping. Crucially, trauma recovery requires ongoing care rather than a one-time intervention. The efficacy of all these approaches is significantly enhanced by actively involving parents and integrating support within community and school structures.
